# Activation of TGF-β-activated kinase 1 (TAK1) restricts *Salmonella* Typhimurium growth by inducing AMPK activation and autophagy

**DOI:** 10.1038/s41419-018-0612-z

**Published:** 2018-05-11

**Authors:** Wei Liu, Yuanyuan Jiang, Jing Sun, Shizhong Geng, Zhiming Pan, Richard A. Prinz, Chengming Wang, Jun Sun, Xinan Jiao, Xiulong Xu

**Affiliations:** 1 Institute of Comparative Medicine, College of Veterinary Medicine, Yangzhou University, Yangzhou, Jiangsu Province 225009 P. R. China; 2 Jiangsu Key Laboratory of Zoonosis, Yangzhou University, Yangzhou, 225009 China; 3 Jiangsu Co-innovation Center for Prevention and Control of Important Animal Infectious Diseases and Zoonosis, Yangzhou University, Yangzhou, Jiangsu Province 225009 China; 4 Department of Surgery, NorthShore University Health System, Evanston, IL 60201 USA; 5 Department of Pathobiology, College of Veterinary Medicine, Auburn University, Auburn, AL 36849 USA; 6 Department of Medicine, University of Illinois at Chicago, Chicago, IL 60612 USA; 7 Department of Cell and Molecular Medicine, Rush University Medical Center, Chicago, IL 60612 USA

## Abstract

Autophagy is a conserved cellular process that functions as a first-line defense to restrict the growth of invading parasitic bacteria. As an intracellular pathogen, *Salmonella (S)* Typhimurium invades host cells through two Type III secretion systems (T3SS) and resides in the *Salmonella*-containing vacuole (SCV). When the SCV membrane is perforated and ruptured by T3SS-1, a small portion of the *Salmonella* egresses from the SCV and replicates rapidly in the nutrient-rich cytosol. Cytosolic *Salmonella* and those residing in the membrane-damaged SCV are tagged by ubiquitination and marked for autophagy through the ubiquitin-binding adaptor proteins such as p62, NDP52, and optineurin. Prior studies suggest that transient intracellular amino-acid starvation and subsequent inactivation of the mechanistic target of rapamycin (mTOR), a key molecule that phosphorylates Unc-51 like autophagy activating kinase (ULK1) and inhibits its activity, can trigger autophagy in *S*. Typhimurium-infected cells. Other studies suggest that energy stress in *S*. Typhimurium-infected cells leads to AMP-activated protein kinase (AMPK) activation and autophagy. In the present study, we report that autophagy was rapidly induced in *S*. Typhimurium-infected cells, as evidenced by increased LC3 lipidation and decreased p62 levels. However, *S*. Typhimurium infection drastically increased AKT phosphorylation but decreased S6K1^T389^, 4E-BP^T37/46^, and ULK1^S757^ phosphorylation, suggesting that mTOR activation by AKT is subverted. Further studies showed that AMPK was activated in *S*. Typhimurium-infected cells, as evidenced by increased ULK1^S317^ and ACC^S79^ phosphorylation. AMPK activation was mediated by Toll-like receptor-activated TAK1. Functional studies revealed that AMPK and TAK1 inhibitors accelerated *S*. Typhimurium growth in HeLa cells. Our results strongly suggest that TAK1 activation leads to AMPK activation, which activates ULK1 by phosphorylating ULK1^S317^ and suppressing mTOR activity and ULK1^S757^ phosphorylation. Our study has unveiled a previously unrecognized pathway for *S*. Typhimurium-induced autophagy.

## Introduction

Autophagy is a highly conserved self-digestion process that plays a crucial role in maintaining cellular homeostasis in response to nutrient depletion or other cellular stresses such as accumulation of damaged organelles, unneeded protein aggregates, and invading microbes^1–4^. Autophagy is controlled by mTOR and AMP-activated protein kinase (AMPK), two nutrient- and energy-sensitive kinases^5^. These two kinases phosphorylate ULK1/2 at different serine residues and have the opposite effect on ULK activity: mTOR phosphorylates ULK1 at serine 757 (ULK1^S757^) and inhibits its activity^5–7^, whereas AMPK phosphorylates ULK1 at multiple sites, including the serine residues 317, 555, and 777, and activates its activity^6–10^. ULK1/2 binds ATG13 and FIP200 proteins to form a preinitiation complex, which controls the activation of the initiation complex that comprises Beclin 1, ATG14L, VPS34, and VSP15^11–13^. VPS34 is a Class III PI-3 kinase and catalyzes phosphatidylinositol (PI)-4,5 to PI-3-phosphate, which initiates the elongation and nucleation of the double membrane to form autophagosomes^4,14,15^.

TAK1 is a member of the mitogen-activated protein kinase kinase kinase family and can be activated by multiple extracellular stimuli such as TGF-β, IL-1, tumor necrosis factor (TNF)-α, and lipopolysaccharide (LPS)^5^. In addition, microbial proteins and the components of host cell signaling pathways can also regulate TAK1 activity^16^. TAK1 phosphorylates and activates several intracellular kinases, including p38, JNK, and I-kappa B kinase complex (IKK). TAK1 plays important roles in cell survival, differentiation, apoptosis, and inflammatory responses^16^. Emerging evidence suggests that TAK1 activation can induce autophagy in an AMPK-dependent manner^17–20^. Whether TAK1 activation by bacterial LPS is responsible for pathogen-induced AMPK activation and autophagy remains to be defined.

*Salmonella spp*. is a facultative intracellular Gram-negative enteropathogen that causes gastroenteritis and typhoid-like fever^21^. *Salmonella* enterica serovar Typhimurium (*S*. Typhimurium) is one of the most common serotypes in human cases of salmonellosis worldwide, despite ongoing implementation of targeted control and prevention measures^22,23^. *S*. Typhimurium invades intestinal epithelial cells and resides in a specialized niche, the *Salmonella*-containing vacuole (SCV)^24^. Damage of the SCV membrane by T3SS-1 enables ~25% *S*. Typhimurium to escape into the cytosol where it can replicate quickly in the nutrient-rich environment. Cytosolic *S*. Typhimurium is ubiquinated to form a dense ubiquitin chain layer on the surface of *Salmonella* that peaks 4 h post invasion^25^. Ubiquitin-decorated *S*. Typhimurium is recognized by multiple autophagy receptors, including NDP52, OPTN, and p62, which bind the LC3-enriched autophore membrane to form autophagosome^26,27^. Recent studies have shown that transient amino-acid starvation due to the cellular membrane damage after *Salmonella* invasion leads to transient AMPK activation and mTOR inactivation, thus triggering the initiation of autophagy^26,28^. Our present study provides evidence that TAK1 activation is responsible for *S*. Typhimurium-induced AMPK activation and autophagy. In addition, we found that TAK1-mediated AMPK activation can subvert the AKT-mediated mTOR activation. Thus, our study provides novel insights how *S*. Typhimurium induces autophagy.

## Results

### Autophagy induction by *S*. Typhimurium in HeLa cells

**Fig. 1:**
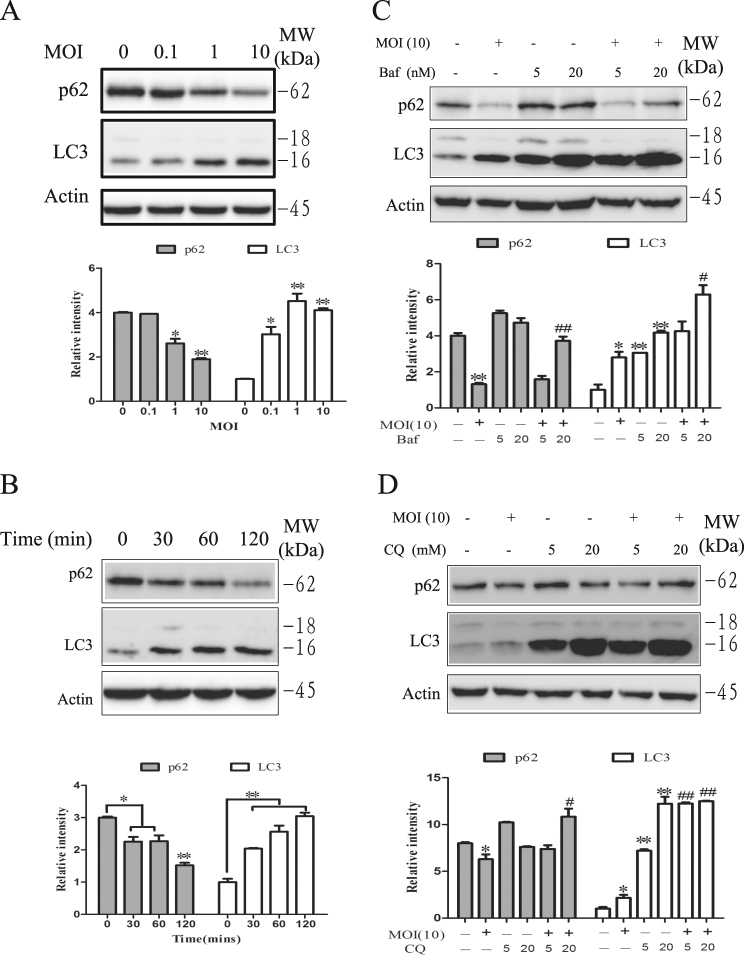
Autophagy induction by *S*. Typhimurium. HeLa cells were infected with indicated MOI of *S*. Typhimurium for 2 h (**a**) or with 10 MOI of *S*. Typhimurium for the indicated lengths of time (**b**). Cell lysates were prepared and analyzed for LC3-II lipidation, p62 and actin by western blot with the indicated antibodies. **c**, **d** Increased LC3-II lipidation was not due to the stall of autophagy flux. HeLa cells pretreated with bafilomycin (Baf; 5 or 20 nM) or chloroquine (CQ; 5 or 20 μM) for 30 min were infected with *S*. Typhimurium (10 MOI) for 2 h. Cell lysates were prepared and analyzed for LC3-II lipidation, p62, and actin by western blot with the indicated antibodies. Blots for LC3-II lipidation, p62, and actin were semi-quantified using NIH Image-J software and presented as bar graphs. Data are presented as the mean ± SD (*n* = 3) relative to control. *p < 0.05, **p < 0.01, compared to uninfected control; ^#^*p* < 0.05, ^##^*p* < 0.01, compared to *S*. Typhimurium infection

**Fig. 2:**
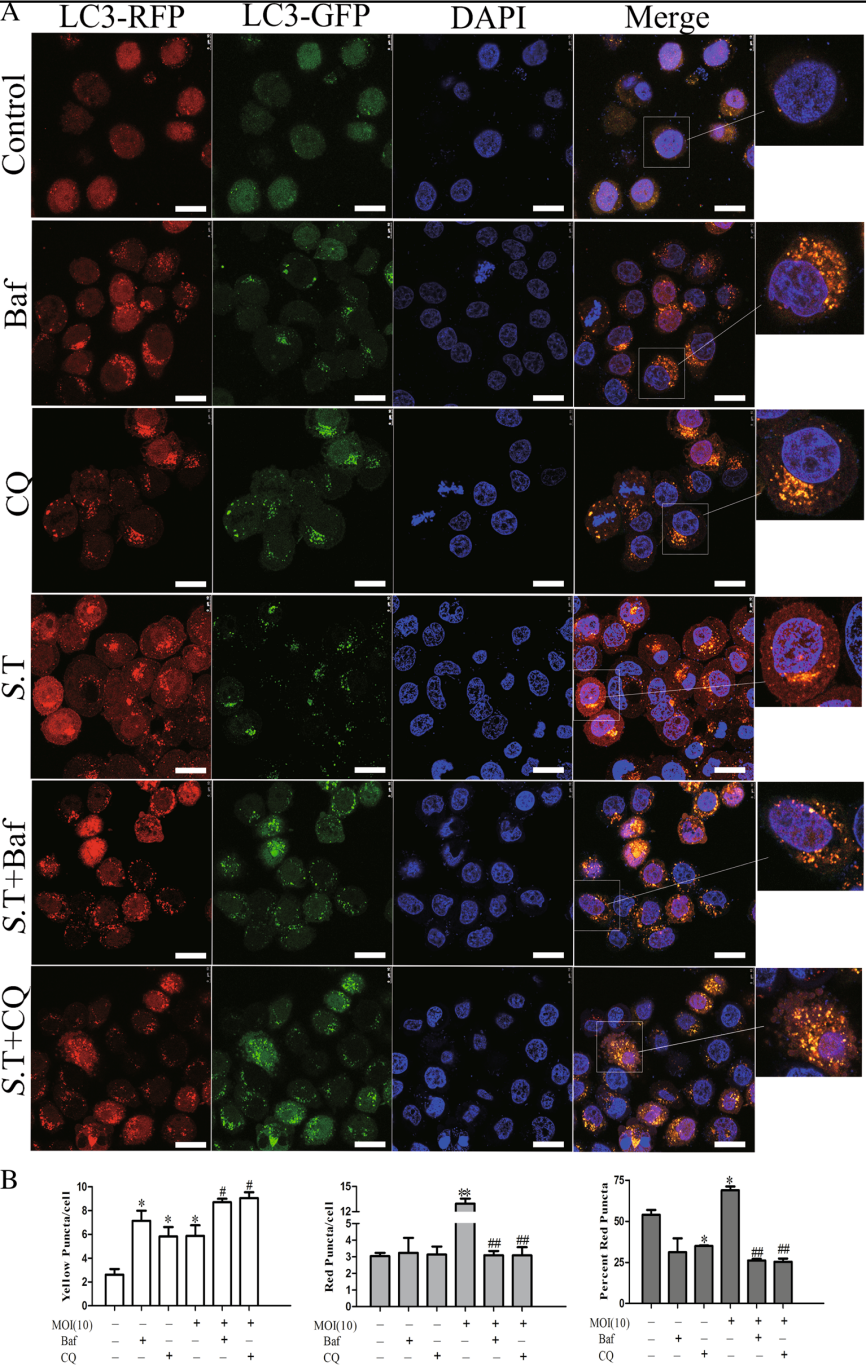
*S*. Typhimurium induces the formation of autolysosomes. HeLa cells stably expressing the GFP-RFP-LC3 gene were infected with *S*. Typhimurium (10 MOI) for 2 h in the absence or presence of bafilomycin (20 nM) or CQ (20 μM). The cells were then fixed and stained with DAPI. Autophagosomes presented as the orange puncta and autolysosomes presented as the red puncta were visualized under a confocal microscope (**a**) and were statistically analyzed (**b**). Bar length: 20 μm. *p < 0.05, **p < 0.01, compared to uninfected control; ^#^*p* < 0.05, ^##^*p* < 0.01, compared to *S*. Typhimurium infection

We first verified the ability of *S*. Typhimurium to induce autophagy in HeLa cells by western blot analysis of LC3 lipidation and p62 degradation. As shown in Fig. 1a,b, *S*. Typhimurium increased LC3-II lipidation and decreased p62 expression in HeLa cells in a dose- (Fig. 1a) and time- (Fig. 1b) dependent manner. Increased LC3-II lipidation was not due to the stall of autophagy flux since combination of *S*. Typhimurium with bafilomycin (5 or 20 nM; Fig. 1c) or chloroquine (CQ; 5 or 20 μM; Fig. 1d) increased the levels of LC3-II and the ratios of LC3-II to LC-I, compared to bafilomycin or CQ alone. Bafilomycin (20 nM; Fig. 1c) or CQ (20 μM; Fig. 1d) partially reversed p62 degradation in *S*. Typhimurium-infected HeLa cells. To further verify the ability of *S*. Typhimurium to induce autophagy, we analyzed the formation of autophagosomes in RFP-GFP-LC3-transfected HeLa cells. As shown in Fig. 2a, there were very few puncta in the uninfected control cells. In contrast, many autophagosomes accumulated in one side of the cytoplasm close to nuclear membrane in *S*. Typhimurium-infected cells. There are average 22 puncta per cell in *S*. Typhimurium-infected HeLa cells (Fig. 2b). Among them, ~70% of the puncta were presented as the red RFP fluorescence dots  (Fig. 2b), suggesting the formation of autolysosomes in which the green GFP fluorescence was quenched under the acidic environments. The number of yellow puncta were also significantly higher in bafilomycin- or CQ-treated cells than in untreated control cells. Among them, the majority of puncta in bafilomycin- or CQ-treated HeLa cells were yellow (Fig. 2b), which means that the red and green GFP fluorescence was merged. Both bafilomycin and CQ significantly increased the number of yellow puncta but decreased the number and percent of red puncta in *S*. Typhimurium-infected cells, suggesting that bafilomycin and CQ block autophagic flux in S. Typhimurium-infected cells.

### Role of mTOR in *S*. Typhimurium-induced autophagy

**Fig. 3:**
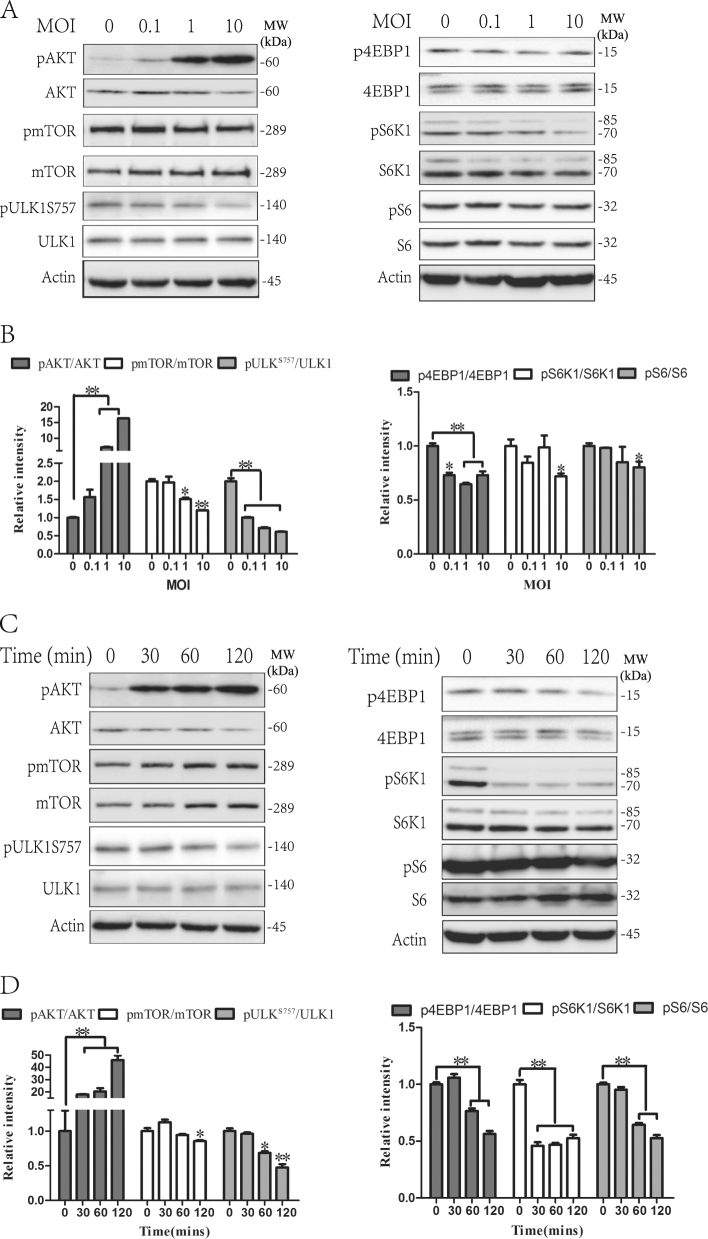
Effect of *S*. Typhimurium on the PI-3 kinase pathway. HeLa cells were infected with the indicated amount of *S*. Typhimurium for 2 h (a) or infected with 2 MOI of *S*. Typhimurium for the indicated length of time (c). Cell lysates were analyzed for AKT, mTOR, ULK1^S757^, 4EBP1, S6K1, and S6 by western blot with the indicated antibodies. Relative phosphorylation levels were analyzed by quantification of the density of the protein bands with NIH Image-J software (b and d) and presented as bar graphs. *p < 0.05, **p < 0.01, compared to uninfected control

Previous studies have established that the sopB protein of *S*. Typhimurium activates AKT^29–32^. mTOR, a downstream effector activated by AKT, suppresses autophagy by phosphorylating ULK1^S757 4,33^. We first analyzed the status of AKT phosphorylation as well as several mTOR substrates, including 4E-BP^T37/46^, S6K1^T389^, and ULK^S757^. *S*. Typhimurium markedly increased AKT^S473^ phosphorylation in a dose- (Fig. 3a & b) and time-dependent (Fig. 3c & d) manner. Unexpectedly, *S*. Typhimurium did not correspondingly increase but rather modestly or weakly decreased the phosphorylation of mTOR^S2448^, 4E-BP^T37/46^, S6^S235/236^, S6K1^T389^, and ULK^S757^ in a dose- (Fig. 3a & b) and time-dependent (Fig. 3c & d) manner. These observations suggest that *S*. Typhimurium can subvert AKT-mediated mTOR activation, and that blocking of mTOR activity may contribute to *Salmonella*-induced autophagy.

### The TAK1–AMPK pathway is activated in *S*. Typhimurium-infected cells

**Fig. 4:**
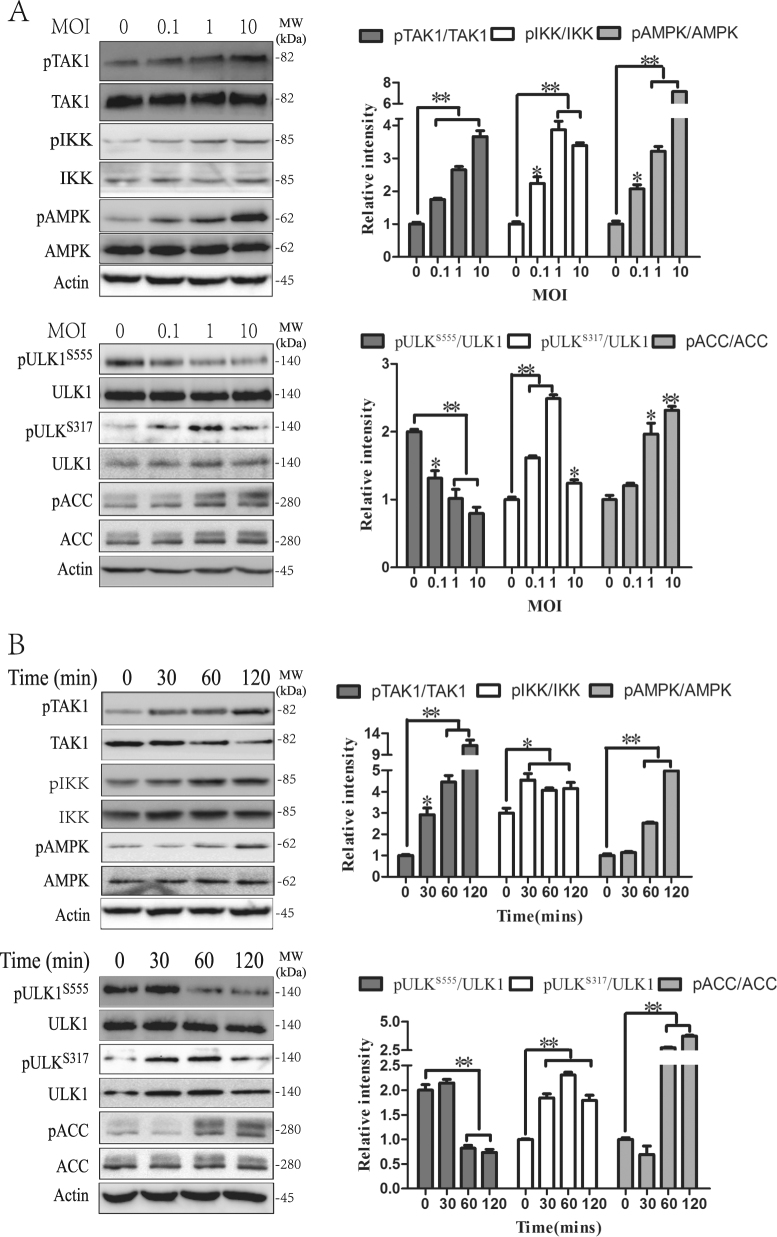
Effect of *S*. Typhimurium on the TAK1–AMPK pathway. HeLa cells were infected with the indicated amount of MOI of *S*. Typhimurium for 2 h (**a**) or were infected with 10 MOI and then incubated for the indicated length of time (**b**). Cell lysates were analyzed for TAK1, IKK, AMPK, ULK1^S555^, ULK1^S317^, and ACC^S79^ by western blot with the indicated antibodies. Relative phosphorylation levels were semi-quantified using NIH Image-J software and presented as bar graphs. Data are presented as the mean ± SD (*n* = 3) relative to control. *p < 0.05, **p < 0.01, compared to uninfected control

It is well established that activation of the Toll-like receptor 4 (TLR4) by LPS leads to TAK1 activation^5^. Emerging evidence suggests that AMPK can be activated by TAK1^17–20^. Here we tested whether *S*. Typhimurium infection could activate TAK1, leading to AMPK activation and autophagy. As shown in Fig. 4, *S*. Typhimurium infection indeed induced TAK1^T184/187^ autophosphorylation in a dose- (Fig. 4a) and time-dependent (Fig. 4b) manner. TAK1 activation led to increased phosphorylation of its two downstream substrates, AMPK^T172^ and IKKα^S176/180^. AMPK phosphorylates multiple serine residues in ULK1^4,14,33^. Interestingly, AMPK activation in *Salmonella*-infected cells increased ULK1^S317^ phosphorylation but decreased ULK1^S555^ phosphorylation in a dose- (Fig. 4a) and time-dependent (Fig. 4b) manner. AMPK activation also led to increased phosphorylation of ACC^S79^, a well-known substrate of AMPK (Fig. 4).

**Fig. 5:**
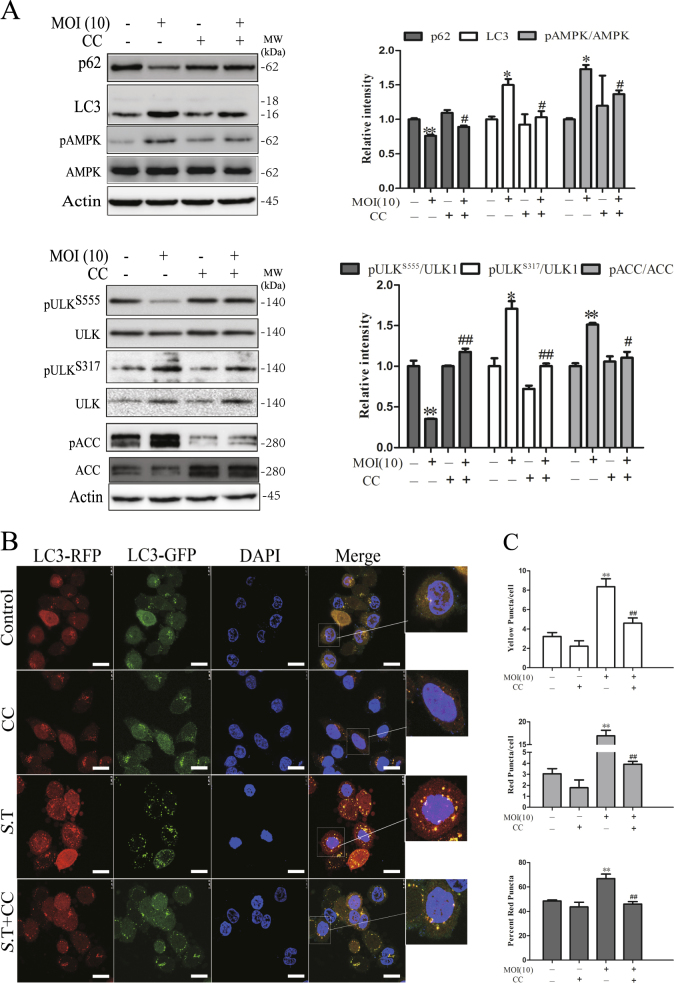
Effect of AMPK inhibitor on *S*. Typhimurium-induced autophagy. HeLa cells pretreated with CC (1 μM) for 30 min were left uninfected or infected with *S*. Typhimurium (10 MOI). After incubation for 2 h, the cell lysates were prepared and analyzed for the levels of p62 and LC3 and for the phosphorylation of AMPK^T172^, ULK1^S555^, ULK1^S317^, and ACC^S79^ (**a**) by western blot with the indicated antibodies or the antibodies against total proteins after stripping. Relative protein and phosphorylation levels were analyzed by quantification of the density of the protein bands with NIH Image-J software and presented as bar graphs. *p < 0.05, **p < 0.01, compared to uninfected control; ^#^*p* < 0.05, ^##^*p* < 0.01, compared to *S*. Typhimurium infection. **b**, **c** CC inhibits *S*. Typhimurium-induced formation of autolysosomes. HeLa cells stably expressing the GFP-RFP-LC3 gene were left uninfected or infected with *S*. Typhimurium (10 MOI) for 2 h in the absence or presence of CC (1 μM). The cells were then fixed and stained with DAPI. Autophagosomes represented by the orange puncta and autolysosomes represented by the red puncta were visualized under a confocal microscope (**b**) and statistically analyzed (**c**). Bar length: 20 μm. *p < 0.05, **p < 0.01, compared to uninfected control; ^#^*p* < 0.05, ^##^*p* < 0.01, compared to *S*. Typhimurium infection

We next determined whether AMPK was indeed responsible for *S*. Typhimurium-induced autophagy and the change of ULK1 phosphorylation. As shown in Fig. 5a, compound C (CC), a specific inhibitor of AMPK, blocked *S*. Typhimurium-induced LC3-II lipidation and p62 degradation. Meanwhile, CC blocked *S*. Typhimurium-induced AMPK^T172^, ULK1^S317^, and ACC^S79^ phosphorylation and reversed the decrease of ULK1^S555^ phosphorylation (Fig. 5a). Consistently, CC blocked the formation of autophagosomes and autolysosomes in *S*. Typhimurium-infected cells (Fig. 5b), as the number of red and yellow fluorescent puncta was significantly lower in *S*. Typhimurium-infected cells in the presence of CC than in the absence of CC. These observations collectively suggest that AMPK activation plays an important role in *S*. Typhimurium-induced autophagy.

### Role of TAK1 in AMPK-mediated regulation of autophagy

**Fig. 6:**
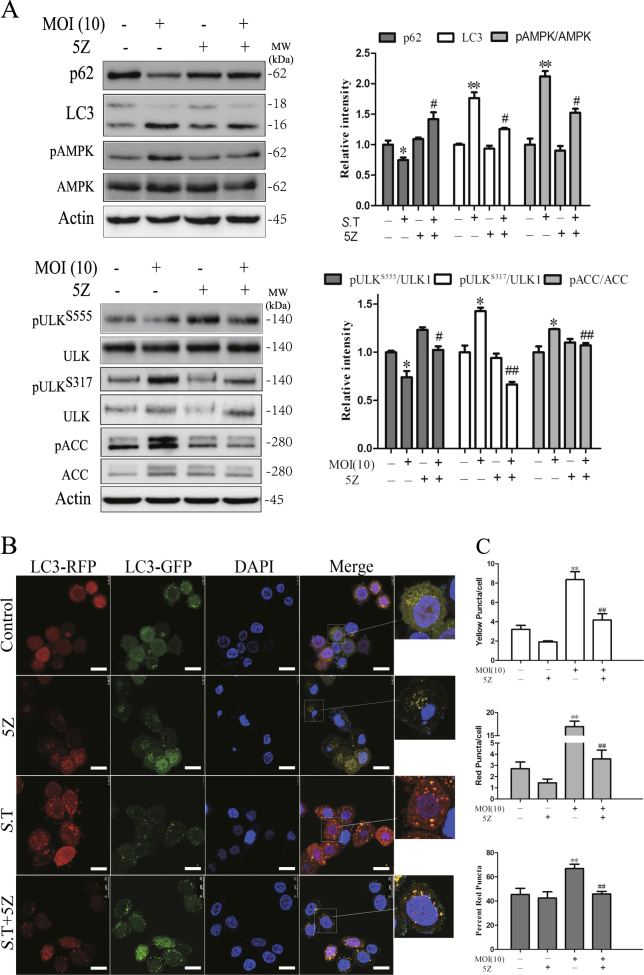
The effect of TAK1 inhibitor on *S*. Typhimurium-induced autophagy. HeLa cells pretreated with 5Z-7-oxozeaenol (5Z; 0.5 μM) for 30 min were left uninfected or infected with *S*. Typhimurium (10 MOI). After incubation for 2 h, the cell lysates were prepared and analyzed for the levels of p62 and LC3 and for the phosphorylation of AMPK^T172^, ULK1^S555^, ULK1^S317^, and ACC^S79^ (**a**) by western blot with the indicated antibodies or the antibodies against total proteins after stripping. Relative protein and phosphorylation levels were analyzed by quantification of the density of the protein bands with NIH Image-J software and presented as bar graphs. *p < 0.05, **p < 0.01, compared to uninfected control; ^#^*p* < 0.05, ^##^*p* < 0.01, compared to *S*. Typhimurium infection. **b**, **c** 5Z inhibits *S*. Typhimurium-induced formation of autolysosomes. HeLa cells stably expressing the GFP-RFP-LC3 gene were left uninfected or infected with *S*. Typhimurium (10 MOI) for 2 h in the absence or presence of 5Z (0.5 μM). The cells were then fixed and stained with DAPI. Autophagosomes represented by the orange puncta and autolysosomes represented by the red puncta were visualized under a confocal microscope (**b**) and statistically analyzed (**c**). Bar length: 20 μm. *p < 0.05, **p < 0.01, compared to uninfected control; ^#^*p* < 0.05, ^##^*p* < 0.01, compared to *S*. Typhimurium infection

We then tested whether TAK1 was responsible for *S*. Typhimurium-induced autophagy and AMPK activation. We first tested whether inhibition of TAK1 activity by 5Z-7-oxozeaenol (5Z), an inhibitor of TAK1, was able to block *S*. Typhimurium-induced autophagy and AMPK activation. As shown in Fig. 6a, 5Z blocked *S*. Typhimurium-induced LC3-II lipidation and p62 degradation, blocked *S*. Typhimurium-induced AMPK^T172^, ULK1^S317^, and ACC^S79^ phosphorylation, and blocked the decrease of ULK1^S555^ phosphorylation (Fig. 6a). Consistently, 5Z blocked the formation of autophagosomes and autolysosomes in *S*. Typhimurium-infected cells (Fig. 6b), as the number of red and orange fluorescent puncta was significantly lower in *S*. Typhimurium-infected cells in the presence of 5Z than in the absence of 5Z (Fig. 6c).

**Fig. 7:**
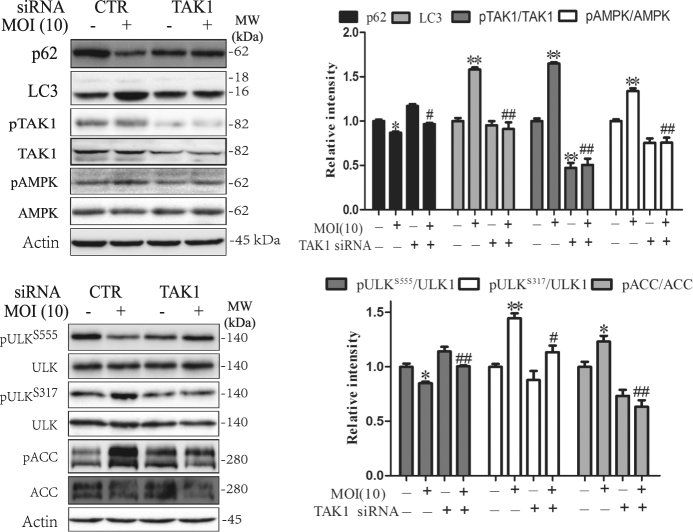
The effect of TAK1 siRNA on *S*. Typhimurium-induced autophagy. HeLa cells were transfected with control or TAK1 siRNA. After incubation for 48 h, the cells were left uninfected or infected with *S*. Typhimurium (10 MOI). After incubation for 2 h, the cell lysates were prepared and analyzed for the levels of p62 and LC3 and for the phosphorylation of AMPK^T172^, ULK1^S555^, ULK1^S317^, and ACC^S79^ by western blot with the indicated antibodies or the antibodies against total proteins after stripping. Relative protein and phosphorylation levels were analyzed by quantification of the density of the protein bands with NIH Image-J software and presented as bar graphs. *p < 0.05, **p < 0.01, compared to uninfected control; ^#^*p* < 0.05, ^##^*p* < 0.01, compared to *S*. Typhimurium infection

The role of TAK1 in mediating *S*. Typhimurium-induced autophagy was further investigated by using TAK1 siRNA. As shown in Fig. 7, TAK1 siRNA effectively suppressed TAK1 expression, and inhibition of TAK1 expression led to the inhibition of *S*. Typhimurium-induced phosphorylation of TAK1^T184/187^, AMPK^T172^, ULK1^S317^, and ACC^S79^. TAK1 siRNA blocked *S*. Typhimurium-induced LC3-II lipidation and p62 degradation, and restored ULK1^S555^ phosphorylation in *S*. Typhimurium-infected HeLa cells (Fig. 7). These observations collectively suggest that TAK1 plays a critical role in mediating *S*. Typhimurium-induced activation of AMPK.

### AMPK activation circumvents mTOR activity

**Fig. 8:**
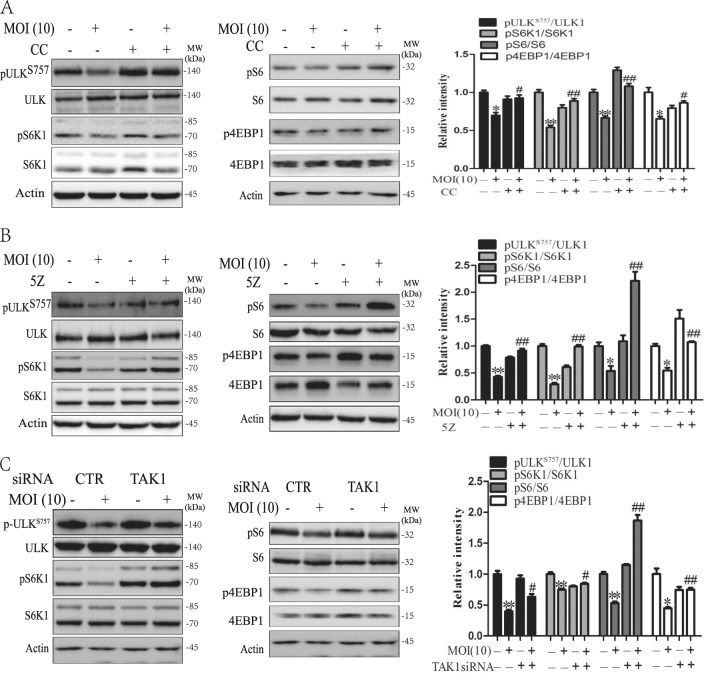
TAK1-medidated AMPK activation circumvents mTOR activity induced by *S*. Typhimurium. HeLa cells were pretreated with CC (1 μM) or 5Z (0.5 μM), or transiently transfected with control or TAK1 siRNA for 48 h. The cells were left uninfected or infected with *S*. Typhimurium (10 MOI). After incubation for 2 h, the cell lysates were prepared and analyzed for the phosphorylation of ULK1S757, S6K1, S6, and 4E-BP (**a**, **c**) by western blot with the indicated antibodies. Relative phosphorylation levels were analyzed by quantification of the density of the protein bands with NIH Image-J software and presented as bar graphs. *p < 0.05, **p < 0.01, compared to uninfected control; ^#^*p* < 0.05, ^##^*p* < 0.01, compared to *S*. Typhimurium infection

It has been well documented that AMPK activation can lead to the suppression of mTOR activity by phosphorylating Raptor^4^. Here we tested whether subversion of AKT-mediated mTOR activation in *S*. Typhimurium-infected HeLa cells was due to TAK1-activated AMPK. As shown in Fig. 8, *S*. Typhimurium modestly or weakly decreased ULK1^S757^, S6K1^T389^, 4E-BP^T37/46^, and S6^S235/236^ phosphorylation, which was blocked by CC (Fig. 8a), 5Z (Fig. 8b), or TAK1 siRNA (Fig. 8c). These observations suggest that AKT-mediated mTOR activation is subverted by TAK1-activated AMPK.

### The effect of autophagy on bacterial invasion and replication

**Fig. 9:**
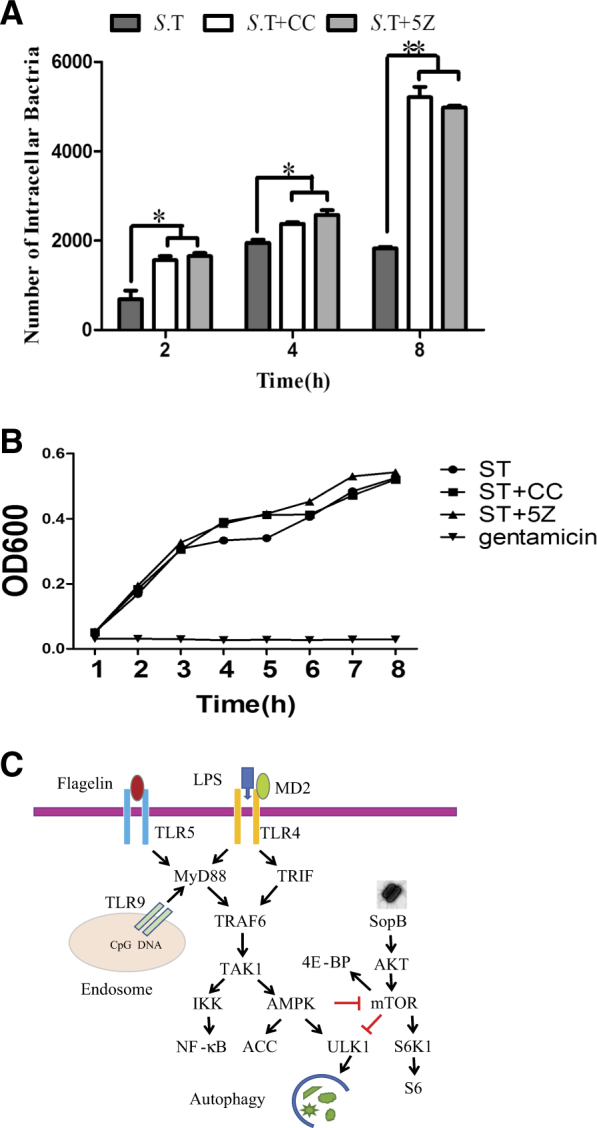
The effect of TAK1 and AMPK inhibitors on *S*. Typhimurium growth. **a** HeLa cells were left untreated or pretreated with CC (1 μM) or 5Z (0.5 μM) for 30 min. The cells were then infected with *S*. Typhimurium (10 MOI). After incubation for the indicated lengths of time, the cells were harvested and lysed. The colony formation units were analyzed by counting the number of bacterial colonies grown in the LB plates. The results represent the mean ± SD from the triplicate from one of three experiments with similar results. *p < 0.05, **p < 0.01, compared to uninfected control. **b** *S*. Typhimurium inoculated in LB medium (100 μl) was cultured in the absence or presence of CC (0.5 μM) or 5Z (1 μM) at 37 °C for 8 h with agitation. The OD600 values of triplicate cultures in LB medium were determined in the indicated intervals of indicated time. Gentamycin (100 μg/ml) was used as a positive control. **c** Schematic mode of *Salmonella*-induced autophagy. The binding of the TLR4-MD2 complex by LPS, which is abundantly present in the wall of the Gram-negative bacteria such as *S*. Typhimurium, activates TRAF6 through its two adaptor proteins, MyD88 and TRIF. In addition, the binding of TLR5 and TLR9 by flagelin and CpG, respectively, can also activates TRAF6 through MyD88. As a E3 ubiquitin ligase, TAFF6 induces TAK1 K63-ubiquitination and activation, leading to NF-kB and AMPK activation, the former regulates the expression of inflammatory cytokines, whereas the latter activates ULK1 and regulates autophagy. AKT activation by the sopB protein of *S*. Typhimurium would activate its downstream effector mTOR and subsequently suppress autophagy. In our model, AMPK activation by TAK1 circumvents the AKT-mediated mTOR activation by phosphorylating Raptor, a subunit in the mTORC1 complex. Inactivation of mTOR activity suppresses ULK1^S757^ phosphorylation and induces ULK1 activation and autophagy

Finally, we tested whether inhibition of the TAK1–AMPK pathway would lead to increased *S*. Typhimurium replication. As shown in Fig. 9a, infection TAK1 inhibitor 5Z and AMPK inhibitor CC significantly increased the number of *S*. Typhimurium in HeLa cells at 2, 4, and 8 h post infection. However, neither 5Z nor AMPK had any effect on the growth of *S*. Typhimurium in vitro in LB medium (Fig. 9b). These observations suggest that the inhibitory effect of 5Z and CC on *Salmonella* replication in HeLa cells is likely mediated by autophagy suppression.

## Discussion

Autophagy cooperates with innate immunity to clear the intracellular bacteria^34,35^. Pattern recognition receptors such as the TLRs can cross-activate the autophagic pathway^36–40^. The underlying molecular mechanisms remain elusive. TAK1 is a serine/threonine kinase activated by numerous inflammatory cytokines and by TLR through the ubiquitin ligase TRAF6 (Fig. 9c)^5^. Although emerging evidence suggests that TAK1 can activate AMPK to induce autophagy^17–20^, whether TAK1 activation by bacteria is responsible for autophagy induction has not been investigated. In the present study, we demonstrated that TAK1 activity was required for AMPK activation and autophagy induction in *S*. Typhimurium-infected HeLa cells. We postulate that the binding of TLR4, TLR5, and TLR9 by LPS, flagellin, and CpG of *S*. Typhimurium, respectively, activates TRAF6 through Myd88 and/or TRIF, leading to TAK1 and AMPK activation. AMPK induces autophagy by activating ULK1 through phosphorylating ULK1^S317^ but blocks ULK1^S757^ phosphorylation by suppressing mTOR1 activity (Fig. 9c). Our study has unveiled a previously unrecognized signaling pathway that plays a central role in *Salmonella*-induced autophagy.

AMPK is a crucial kinase in autophagy regulation. AMPK senses the intracellular energy state^4^. When the ratios of intracellular AMP/ATP levels are increased, AMPK is then activated. In addition, AMPK can be activated by three protein kinases, including LKB1, calcium/calmodulin-dependent kinase kinase, and TAK1^4,14,33^. AMPK activates ULK1 by phosphorylating ULK1 at multiple serine residues, including S317, S555, S777, and S468^4,14,33^. In addition, AMPK can indirectly activate ULK1 by suppressing mTOR activity and decreasing ULK1^S757^ phosphorylation^41–43^. A recent study by Ganesan et al.^44^. reported that the *S*. Typhimurium SL1344 transiently activates AMPK activity 1 h post infection in mouse bone marrow-derived macrophages by decreasing the ATP levels. Further studies demonstrated that AMPK along with its upstream effectors, LKB kinase and SIRT1 deacetylase, are re-located into the membrane of autolysosomes where they are degraded by lysosomal proteases. AMPK downregulation thus restrains the sustained AMPK activation and autophagy, as evidenced by increased LC3 lipidation and decreased p62 levels, which only transiently occur ~1 h after *S*. Typhimurium infection^44^. Our present study demonstrated that increased AMPK phosphorylation and LC3-II lipidation as well as decreased p62 levels were maintained in *S*. Typhimurium-infected HeLa cells for at least 2 h. In addition, AMPK protein levels were not decreased during this time period. It is not clear whether the difference in AMPK activation and autophagy kinetics in *S*. Typhimurium-infected bone marrow-derived macrophages and HeLa cells is due to the different cell types. Nevertheless, both studies suggest that AMPK activation plays a critical role in *S*. Typhimurium-induced autophagy.

The TLRs have been implicated in regulating autophagy^35,38,40^. For example, activation of TLR4 and TLR3 by LPS and polyinosinic–polycytidylic acid, respectively, induced autophagy in macrophages and lung cancer cells^37,45^. A recent study by McCarthy et al. showed that the TAK1–AMPK pathway is activated by TLR9 in vascular smooth muscle cells^46^. Several earlier studies indicate that TLR4 activation by LPS, which is abundantly present on the cell walls of Gram-negative bacteria, induces autophagy through TRIF-mediated disruption of the Bcl-2-Beclin1 interaction^37,45^. Although it has been long recognized that the availability of Beclin1 alone is not sufficient to trigger autophagy, what other signaling pathways activated by TLR lead to autophagy induction remains largely uncharacterized^35,38,40^. TLR4 activation by LPS activates NF-κB through TAK1-mediated IKK activation (Fig. 9c)^4,5^. In addition to activating IKK, TAK1 can also activate AMPK by phosphorylating T172, which subsequently induces autophagy^4^. For example, Ding et al.^47^ reported earlier that TAK1 is required for TGF-β-induced autophagy in murine mesangial cells. Herrero-Martin et al.^18^ reported that TNF-related apoptosis-inducing ligand (TRAIL) induces autophagy in human epithelial cells by AMPK in a TAK1-dependent and LKB1-independent manner. Xie et al. reported that AMPK activation is blocked in TAK1-deficient mouse embryos and mouse fibroblasts^20^. TAK1 activates AMPK-dependent cytoprotective autophagy in TRAIL-treated epithelial cells^18^. TAK1 is responsible for VEGF–induced AMPK activation in endothelial cells^48^. We recently reported that TAK1 is also responsible for S6K1 inhibition-induced AMPK activation and autophagy^49^. Our present study showed that TAK1 was activated in *Salmonella*-infected HeLa cells in a time- and dose-dependent manner, and that TAK1 activation led to increased IKKα and AMPK phosphorylation. We further demonstrated that TAK1 siRNA and 5Z blocked *Salmonella*-induced AMPK activation and autophagy. These observations collectively suggest that TAK1 plays a critical role in *S*. Typhimurium-induced AMPK activation and autophagy.

The binding of TLR2/1, TLR2/6, TLR4, TLR5, and TLR9 by diacyl lipoproteins, triacyl lipoproteins, LPS, flagelin, and CpG, respectively, can readily activate TAK1^50^. It is noteworthy that TAK1-mediated AMPK activation in *S*. Typhimurium-infected HeLa cells did not increase ULK1^S555^ but rather increased ULK1^S317^ phosphorylation. This observation concurs with a previous study showing that ULK1 is phosphorylated at S317 and S777 but not at S555 in HEK293 cells under glucose starvation^6^. Interestingly, ULK1^S555^ phosphorylation was increased in *Salmonella*-infected RAW264.7 cells, a murine macrophage cell line (Liu et al., unpublished observations). Ganesan et al.^44^ reported that *S*. Typhimurium infection of bone marrow-derived macrophages causes energy depletion, as evidenced by decreased intracellular ATP levels. Macrophages are very sensitive to *S*. Typhimurium killing due to necroptosis and pyroptosis. We did not analyze intracellular AMP and ATP levels in *S*. Typhimurium-infected HeLa cells. It is not clear whether decreased energy levels may also contribute to AMPK activation in HeLa cells.

mTOR plays a critical role in regulating autophagy^51^. mTOR phosphorylates ULK1^S757^ and inhibits its activity, leading to the suppression of autophagy^4,33^. Absence of ULK1^S757^ phosphorylation under nutrient depletion leads to ULK1 activation and autophagy induction^4,33^. It is well established that sopB of *S*. Typhimurium activates AKT in both macrophages and in epithelial tumor cell lines^29–32^. In addition, AKT can be targeted by *S*. Typhimurium to the SCV membrane of peritoneal exudate macrophages and activated by FAK^52^. Thus, AKT/mTOR activation has been considered a mechanism by which *Salmonella* evades autophagy-mediated destruction of intracellular bacteria^52^. Tattoli et al.^53^ reported that AKT is activated in HeLa cells; it is not clear whether mTOR is correspondingly activated since phosphorylation is decreased in one of its substrates, 4E-BP, but is increased in another substrate, S6K1, as well as S6. Although the status of ULK1^S757^ is not known, these investigators suggest that transient amino-acid starvation leads to the suppression of mTOR activity^53^. Genesan et al. reported that phosphorylation of AKT and S6K1, a downstream substrate of mTOR, is persistently increased in *S*. Typhimurium-infected bone marrow-derived macrophages^54^. In the present study, we demonstrated that, although AKT phosphorylation was remarkably increased in *S*. Typhimurium-infected HeLa cells in a dose- and time-dependent manner, several substrates of mTOR, including S6K1^T389^, 4E-BP^T37/46^, and ULK1^S757^, were not correspondingly increased but rather modestly or weakly decreased in a dose- and time-dependent manner. On the basis of the well-established negative regulation of mTORC1 by AMPK phosphorylation of Raptor^55,56^, we propose that blockade of mTOR activation in *S*. Typhimurium-induced HeLa cells is due to the inhibition of mTORC1 activity by AMPK. Indeed, increased phosphorylation of S6K1^T389^, ULK1^S757^, and 4E-BP^T37/46^ was detected in *S*. Typhimurium-infected HeLa cells in the presence of either a TAK1 or AMPK inhibitor as well as TAK1 siRNA. These observations suggest that AMPK activation by TAK1 can subdue AKT-mediated mTOR activation, leading to decreased ULK1^S757^ and S6K1^T389^ phosphorylation in *S*. Typhimurium-infected HeLa cells (Fig. 9c).

Autophagy plays an important role in restricting *S*. Typhimurium bacterial growth in epithelial cells and macrophages. *S*. Typhimurium grows faster in ATG4-deficient murine embryonic fibroblast cells than in the wild-type cells^57^. Optineurin is an important autophagic receptor in *S*. Typhimurium invasion. The colonization rate of *S*. Typhimurium is twofold higher in the OPTN-deficient cells^58^. Genetic inactivation of the autophagic pathway by ATG1, ATG6, and ATG7 gene knockout increases intracellular bacterial replication and decreases the lifespan of two model organisms, *Caenorhabditis elegans* and *Dictyostelium disodium*^59^. Consistently, there are 100-fold more *S*. Typhimurium bacteria in the mesangial lymph nodes and spleen of the mice with ATG16L1 conditionally knocked out in the intestinal epithelial cells than in that of wild-type mice^60^. Trifluoperazine, an autophagy activator, inhibits the replication of *S*. Typhimurium in HeLa cells^60^. *S*. Typhimurium bacterial burdens are ~100-fold heavier in the spleen and liver of mice, with ATG5 being conditionally knocked out in the intestinal epithelial cells^61^. Our present study showed that AMPK and TAK1 inhibitors, both of which inhibited *S*. Typhimurium-induced autophagy, accelerated bacterial growth in HeLa cells, suggesting that autophagy can restrict intracellular bacterial growth.

We are aware of several weaknesses in our current study. First, while HeLa cells have been widely used as a model system for studying the mechanisms of *Salmonella*-induced autophagy, the results might be different in the relevant cell types of *Salmonella* infection such as macrophages and intestinal epithelial cells. Secondly, the role of TAK1 in mediating *Salmonella*-induced AMPK activation and autophagy and in restricting bacterial growth was not investigated in vivo in TAK1-deficient mice. These in vitro observations need to be verified in vivo in a mouse model. Thirdly, while prior extensive evidence suggests that TAK1 is activated through TLR4, the role of TLR4 as well as its adaptor proteins such as MyD88 and TRIF in mediating *Salmonella*-activated TAK1–AMPK pathway was not investigated in the present study. Data from these detailed studies should further strengthen our conclusion.

In summary, our present study has provided unambiguous evidence that AMPK is activated in *S*. Typhimurium-infected HeLa cells, and that TAK1, a kinase activated by multiple TLRs such as TLR4 by LPS, TLR5 by flagellin, and TLR9 by CpG, is primarily responsible for AMPK activation (Fig. 9c). Activated AMPK phosphorylates ULK1^S317^ and meanwhile circumvents AKT-mediated mTOR activation, leading to decreased ULK1^S757^ phosphorylation. Thus, ULK1 is activated through increased phosphorylation at S317 and decreased phosphorylation at S757. ULK1 activation plays an important role in the autophagy initiation in *Salmonella*-infected cells. Our study establishes a previously unrecognized link between the TLR signaling and autophagic pathways (Fig. 9c).

## Materials and methods

### Reagents

Bafilomycin, CQ, and 5Z were purchased from Sigma (St. Louis, MO). CC was purchased from Selleck Inc. (Houston, TX). Anti-actin mAb was purchased from Santa Cruz Biotechnology Inc. (Santa Cruz, CA). Antibodies against LC3, ULK1, AMPK, mTOR, AKT, S6K1, 4E-BP, S6, ACC (acetyl-CoA carboxylase), TAK1, and their corresponding phospho-antibodies including ULK1^S555^, ULK1^S757^, ULK1^S317^, AMPK^T172^, mTOR^S2448^, AKT^S473^, S6K1^T389^, S6^S235/236^, 4E-BP^T37/46^, IKKα^S176/180^, ACC^S79^, and TAK1^T184/187^ were purchased from Cell Signaling Technology (Danvers, MA).

### Cells

HeLa cells were purchased from American Tissue Culture Collection (Manassas, VA). The cells were grown in the complete DMEM medium supplemented with 10% fetal bovine serum, streptomycin and penicillin, and L-glutamine. HeLa cells infected with a lentiviral vector encoding the pLV-LC3-GFP-RFP gene (Chengdu Transvector Biotechnology Inc., Chengdu, China) were selected in the complete medium containing puromycin (1.5 μg/ml; Life Technologies).

### Bacteria

*S*. Typhimurium wild-type strain SL1344 was obtained from the National Institute for the Control of Pharmaceutical and Biological Products (NICPBP), China. Bacteria were grown nonagitated in 10 ml of Luria-Bertani (LB) broth with 0.01 ml of a stationary-phase culture, followed by overnight incubation (>18 h) at 37 °C^62^.

### Infection of cells

HeLa cells seeded in six-well plates were infected with the indicated multiplicity of infection (MOI) of *S*. Typhimurium (SL1344). After 30 min, extracellular bacteria were removed. The cells were incubated for 30 min in the medium containing 100 μg/ml of gentamicin and then were washed and subsequently cultured in the medium containing gentamycin (10 μg/ml) for the indicated length of time. The cells were harvested and analyzed by western blot with the indicated antibodies.

### Western blot

Cells grown in six-well plates were harvested and lysed in NP-40 lysis buffer (50 mM Tris-HCl (pH 8.0), 150 mM NaCl, 1% NP-40, 5 mM EDTA, 10 µg/ml aprotinin, 10 µg/ml leupeptin, and 1 mM phenylmethylsulfonyl fluoride). After incubation on ice for 30 min, the cell lysates were prepared by spinning down at 4 °C, 15,000 r.p.m. for 15 min. Cell lysates were analyzed by western blot with antibodies against the proteins of interest, followed by horseradish peroxidase-conjugated goat anti-rabbit IgG and SuperSignal Western Pico enhanced chemiluminoscence substrate (Pierce Chemical Co., Rockford, IL). The density of the bands was analyzed by using NIH Image-J software and normalized by the arbitrary units of their corresponding total proteins or β-actin as indicated. Quantified results were presented as the mean ± SD from three experiments in bar graphs.

### TAK1 knockdown

TAK1 siRNA was purchased from Cell Signaling Technology (Danvers, MA). A scrambled control siRNA was purchased from Life Technologies (Invitrogen Life Technologies, Grand Island, NY). HeLa cells seeded in a six-well plate were transfected with siRNA using Lipofectamine RNAiMAX (Invitrogen Life Technologies) according to the manufacturer’s instruction. After incubation for 48 h, the cells were left uninfected or infected with 10 MOI and then incubated for 2 h. The cell lysates were prepared and analyzed for the expression of TAK1 and other relevant proteins.

### Bacterial colonization

To determine the effect of CC and 5Z-7-oxozeaenol on *S*. Typhimurium colonization in LB, *S*. Typhimurium was prepared as described above. An aliquot of 100 μl bacterial cultures were grown in liquid LB medium, or LB with CC (0.5 μM) /5Z-7-oxozeaenol (1 μM), at 37 °C for 8 h with agitation. The OD600 values of triplicate cultures in LB medium were determined in 1-h intervals. To ascertain bacterial invasion, RAW264.7 and HeLa cells were seeded in 24-well plates, and the cells were infected with *S*. Typhimurium as described above. Cells were incubated for an additional 120 min in DMEM with gentamicin, washed, and incubated with shaking in HBSS containing Triton X-100 in a cold room. Bacterial CFU were determined by plating diluted cell lysates onto MacConkey agar culture plates (Difco Laboratories Inc.) and incubating the cultures at 37 °C overnight.

### Autophagosome analysis

HeLa cells stably transfected with GFP-RFP-LC3 were seeded on coverslips. After infection of *S*. Typhimurium, the cells were incubated in the absence or presence of CC (1 μM) or 5Z-7-oxozeaenol (0.5 μM). After incubation for 2 h, the cells were fixed in 4% paraformaldehyde at room temperature for 10 min. The coverslips were mounted with 50% glycerin in PBS 4,6-diamidino-2-phenylindole (0.5 μg/ml; Sigma Chemical Co.). Autophagosomes were examined under a Leica LP8 confocal microscope. The red and orange puncta in the cells of 10 random fields (100×) were counted in a blinded manner. Results represent the mean puncta per cell ± SD from one of three independent experiments with similar results. Percent red puncta = the number of red puncta ÷ (the number of red puncta + the number of orange puncta) × 100%

### Statistical analysis

All statistics was performed with SigmaPlot 11 software (Systat Software Inc, San Jose, CA). The differences in the number of puncta in HeLa cells and the density of scanned bands were statistically analyzed by using an unpaired Student's *t-*test. A *p* value of <0.05 was considered statistically significant.
